# Therapeutic Alliance and In-Session Therapy Needs of Male Adult Sexual Assault Survivors: Perspectives of Survivors and Therapists

**DOI:** 10.1007/s10508-025-03172-9

**Published:** 2025-06-19

**Authors:** Michal Guter, Tomer Einat, Keren Gueta

**Affiliations:** 1 Department of Criminology, Bar-Ilan University, Anna & Max Webb, 52900 Ramat-Gan, Israel; 2 School of Criminology, Haifa University, Haifa, Israel

**Keywords:** Adult sexual assault, Masculinity, Male victimization, Therapeutic alliance, Gender-sensitive therapy

## Abstract

Male sexual victimization is more commonly examined in the context of child sexual abuse than adult sexual assault (ASA; 18+), with therapeutic research largely centering on female survivors, who constitute the main focus of scholarly attention. Consequently, this study explored how the therapeutic alliance is built and what in-session therapy needs emerge for male ASA survivors, as shaped by masculinity and adulthood. Data were collected from 39 in-depth interviews with Jewish Israeli participants, comprising two distinct groups: 19 male ASA survivors and 20 sexual trauma therapists. The data were analyzed separately through reflexive thematic analysis, and only at the final stage were the two groups examined together. Three key findings were identified: (1) facilitators of therapeutic alliance with male ASA survivors include awareness of how age and masculinity shape their experiences, psychoeducation for bonding, and present-focused listening; (2) barriers to therapeutic alliance include an overemphasis on the traumatic event itself, with a therapeutic focus that prioritizes trauma details at the expense of attending to the survivor’s broader identity, current needs, and lived experiences; and (3) in-session therapy needs include psychoeducation, present-focused therapy, and changing the therapeutic setting (e.g., stepping out of the therapy room or going for walks). Accordingly, we conclude that the therapeutic alliance and in-session therapy needs of male ASA survivors are shaped by the dual lenses of age and masculinity, highlighting the necessity of adapting therapeutic approaches to address these factors.

## Introduction

Despite growing recognition of male victimization (Depraetere et al., 2020), limited attention has been given to the age of the male survivor at the time of victimization. While much of male sexual victimization occurs during childhood (child sexual abuse; CSA), data suggest that adult sexual assault (ASA), defined as any non-consensual sexual act experienced by a man aged 18 or older (Peterson et al., 2011), is a growing issue (Depraetere et al., 2020; Smith et al., 2018, 2022). For instance, the National Intimate Partner and Sexual Violence Survey (NISVS) reports that about 44% of rape cases (completed or attempted) occur after the age of 18 (Smith et al., 2018, p. 17). Similarly, in Israel, a recent report by the Association of Rape Crisis Centers in Israel (2024) notes that 13% of annual inquiries were made by male survivors, 30% of whom were adults at the time of victimization. Regardless of this recognition, sexual victimization among men is often assessed using narrow definitions that focus on victim penetration (Depraetere et al., 2020; Smith et al., 2017). This assessment excludes the category of “forced to penetrate” (FTP)—defined as the act in which a male victim is forced to penetrate the perpetrator’s vagina, anus, or mouth using his penis, without his consent (Weare, 2018). Including FTP cases in the NISVS significantly increases the estimated number of male sexual assault (SA) survivors in the United States—from 219,000 (classified as rape) to 1,715,000 when FTP cases are included (Smith et al., 2017).

The growing issue of male victimization in adulthood warrants a dedicated study focused on this population for several reasons. First, the experiences of CSA and ASA differ significantly in both their characteristics (Moreno, 2013) and outcomes (Campbell et al., 2009) of the SA. Second, adulthood is recognized as a distinct life phase, defined by responsibility, independence, and self-sufficiency (Hill et al., 2023; Turanovic & Biro, 2023; Wood et al., 2017). Moreover, the need for such research is further underscored by increasing evidence of the prevalence of male ASA and its severe long-term consequences, including shame, depression, posttraumatic stress disorder (PTSD), and suicidal ideation (Depraetere et al., 2020; Langdridge et al., 2023). Yet, their therapeutic experiences and needs have been largely overlooked, especially compared to the extensive research on female SA survivors (Miles et al., 2024; O’Doherty et al., 2023).

A foundational element of therapy for SA survivors, regardless of gender, is therapeutic alliance, which refers to the holistic and collaborative elements of the therapist-client relationship (Flückiger et al., 2018). The therapeutic alliance comprises a mutual agreement on the therapy goals and tasks, and the bond between the client and therapist (Howard et al., 2022). Studies have underscored its crucial effect, consistently showing a strong link between early alliance formation and positive treatment outcomes (Baier et al., 2020). Studies on therapeutic alliance with men indicate that in order to facilitate it, therapists should normalize and validate men’s experiences to overcome shame and stigma (Bedi & Richards, 2011), use direct and jargon-free language (Seidler et al., 2018; Tyler, 2021), address their own gender assumptions, and consider the impact of masculinity (Kilimnik & Meston, 2021; Seidler et al., 2018). Yet, no study, to the best of our knowledge, has examined the formation of the therapeutic alliance with male ASA survivors. Accordingly, the present study addresses the alliance male ASA survivors form and their in-session therapy needs and does so from the perspectives of both male ASA survivors and sexual trauma therapists.

### Hegemonic Masculinity and Therapy with Male Adult Sexual Assault Survivors

The existing literature on therapy for SA survivors generally suggests several evidence-based trauma-focused therapies for treating symptoms, including psychodynamic psychotherapy, eye movement desensitization reprocessing, cognitive behavioral therapy (CBT), and trauma-focused CBT (Miles et al., 2024; O’Doherty et al., 2023). To date, there is no specific treatment tailored for male ASA survivors, as the topic remains under-researched and the phenomenon itself is considered uncommon (Depraetere et al., 2020). However, two case studies did address this topic. Willis (2009) presented a case study of a male ASA survivor and suggested that effective strategies for coping with shame, depression, isolation, and trauma, should include CBT and support from professionals and loved ones. Similarly, Abbas and MacFie (2013) studied a male ASA survivor who experienced PTSD and dissociative symptoms. Their findings demonstrated that psychodynamic psychotherapy effectively reduced both. Yet, given the small samples and one-sided data, these studies’ recommendations remain anecdotal and their effectiveness for the broader male ASA survivor population is unclear.

While male and female ASA survivors may experience similar trauma, their ways of coping differ due to societal gender expectations (Elkins et al., 2017). For men, these expectations are shaped by the concept of hegemonic masculinity—a set of norms, values, and practices that emphasize strength, capability, aggressiveness, dominance, stoicism, and resilience (Connell, 2005; Connell & Messerschmidt, 2005; King et al., 2021; Levant et al., 2013). Given that SA directly challenges these ideals, male survivors often experience intense stigma and a sense of emasculation, as they may be viewed as weak, powerless, or even feminized (Einat & Guter, 2024; Petersson & Plantin, 2019; Ralston, 2020; Vandello et al., 2008). These dynamics not only shape how men interpret their experiences but also how they engage, or struggle to engage, with traditional therapeutic models, which may feel emotionally exposing or incongruent with masculine norms (Bedi & Richards, 2011; Gueta et al., 2019; Rapsey et al., 2020). Consequently, there is a growing call in the literature to adopt more flexible and responsive therapeutic practices for male survivors’ needs (Bedi & Richards, 2011; Gamache et al., 2025; Rapsey et al., 2020), such as loosening rigid boundaries (Moor et al., 2022) or incorporating peer-led, community-based formats, such as support groups (Konya et al., 2025) to create safer and more accessible spaces for therapy.

Hegemonic masculinity and its associated norms have been found to generate gender-based therapy barriers for men (Gueta & Shlichove, 2022; Mahalik et al., 2012). These include internal obstacles, such as men’s difficulty in discussing their feelings (Hogan, 2022), and external ones, such as services that are unprepared or unwilling to support men (Javaid, 2020). Those barriers may deter men from seeking therapy or result in higher dropout rates (Bedi & Richards, 2011; Rapsey et al., 2020) as well as complicate their ability to engage in key aspects of therapy such as forming a therapeutic alliance (Bedi & Richards, 2011; Seidler et al., 2018). Additionally, many male survivors struggle to recognize or label their experiences as sexual victimization, further complicating their path to recovery (PettyJohn et al., 2022; Ralston, 2020).

Masculinity norms play a significant role in the therapeutic setting. For example, Sherrill (2020) examined Latino male sexual victimization and highlighted how the masculinity form of machismo—associated with aggression and power—shaped survivors’ experiences and needed to be addressed in therapy. The context of masculinity and therapy in Israeli culture, as a non-English-speaking country sharing many attributes with Western masculinity ideals (Gueta & Shlichove, 2022; Lowenstein-Barkai, 2021) warrants deeper exploration. Two key cultural elements—mandatory military service and familism—shape hegemonic masculinity in Israel. Military service idealizes the combat soldier as the epitome of heroism and manhood (Levy & Sasson-Levy, 2008), while familism emphasizes the primacy of close familial bonds and support (Gueta & Shlichove, 2022; Gueta et al., 2025). The hypermasculine military culture equates masculinity with power and control and emphasizes emotional suppression, dominance, and competitiveness, fostering an anti-vulnerability culture that discourages help-seeking after sexual victimization (Gamache et al., 2025; Schaefer et al., 2021).

### The Present Study

Drawing from the aforementioned literature, the present study explores two interrelated dynamics of therapy with male ASA survivors, considering age at the time of victimization and gender as key factors shaping those dynamics. Specifically, it examines how the therapeutic alliance is formed and sustained, and what in-session therapy needs are perceived as essential for effective therapeutic work. By therapy needs, we refer to the therapeutic conditions or adjustments that support male survivors’ ability to engage, process emotions, and remain present during sessions.

To address these aims, the study draws on in-depth interviews with male ASA survivors and sexual trauma therapists, bringing together personal lived experiences and clinical perspectives. This qualitative approach seeks to generate a deeper understanding of the therapeutic challenges and in-session therapy needs that arise when working with men who have been sexually victimized in adulthood. In line with these objectives, we have posited the following research questions: (1) What are the facilitators of the therapeutic alliance with male ASA survivors, and how can they be implemented during therapy? (2) What are the unique in-session therapy needs of male ASA survivors according to the survivors and sexual trauma therapists?

## Method

### Participants

This study included 39 participants who were divided into two groups (19 male ASA survivors and 20 sexual trauma therapists). This sample size was determined in accordance with Braun and Clarke’s (2022) principle that sample size should align with the purpose, scope, and context of the research, as well as the richness of the data. Given the dual focus on survivors and therapists, a larger sample was necessary to capture diverse perspectives. Comparable studies (Sherrill, 2020; Widanaralalage et al., 2023) included 10 and 12 therapists, respectively, and 21, 19, and 9 survivors (Elder et al., 2017; Ralston, 2020; Widanaralalage et al., 2022), respectively, situating this study within established norms. The sample size ensured sufficient breadth and depth for meaningful thematic analysis while remaining manageable for detailed exploration (Braun & Clarke, 2022).

The first group was comprised of 19 cisgender Jewish-Israeli male ASA survivors who were sexually assaulted when over 18 (survivor participants; SPs). At the time of the study, their ages ranged from 22 to 56 (M = 34.8, SD = 9.2); their ASA ages ranged from 18 to 36 (M = 23.9, SD = 5.4). Sixteen participants reported engaging in therapy at some point after the ASA.^1^ Participants had diverse occupational backgrounds, including psychotherapy (3), teaching (2), office work (2), editing (1), gardening (1), and volunteering (1). A significant portion were students (6), and three participants were not employed at the time of the study. Of these 19 participants, 11 were raped (four by FTP), and eight others were sexually assaulted. 11 were assaulted by men, and eight by women. 18 self-identified as heterosexual, and one as bisexual.

The inclusion criteria for SP participants required that they had experienced ASA, which in this study was defined as unwanted sexual contact, rape, or FTP incidents occurring after the age of 18 and perpetrated by either gender using verbal pressure, exploitation, physical force, or intoxication (Peterson et al., 2011; Smith et al., 2018). However, participants who had also experienced CSA were included, given research indicating that CSA can increase the risk of ASA through revictimization (see Charak et al., 2019). As a result, eight out of the 19 SPs had experienced both CSA and ASA.

This sample reflected the ethnic diversity within the Jewish Israeli population: 11 participants were second-generation Ashkenazi (European Jewish immigrants), seven were second-generation Mizrahi (North African and Middle Eastern Jewish immigrants), and one participant was from a second-generation family originating from the former Soviet Union (see Levy & Sasson-Levy, 2008).

The second participant group included 20 therapists (therapist participants; TPs; 13 males, seven females) specializing in sexually assaulted men. Their years of experience as sexual trauma therapists ranged from 5 to 30 (M = 12.9, SD = 6.2). Two had doctoral degrees in social work; 15 had graduate degrees (in clinical social work (7), clinical criminology (3), social work (2), art therapy (2), and educational counseling (1)); three participants held specialized certifications: one in psychodynamic psychotherapy for the treatment of sexual trauma, one in trauma-focused psychotherapy, and one in group therapy and consulting. The participation criteria required TPs to have a minimum of five years of experience working with male ASA survivors and to have treated at least three male ASA survivors in their practice. These criteria were established to ensure participants possessed adequate professional familiarity with male ASA survivors (Barnard, 2018; Paul & Paul, 2016). To ensure participants met inclusion criteria, they provided a summary of their professional background during a preliminary phone call, including years of experience, specialization, and work with male ASA survivors. Those who qualified were invited to an interview to elaborate on their experience and therapeutic approach. This two-step process ensured that all participants had the relevant expertise and practical knowledge required for the study.

### Procedure

Members of the SPs group were recruited by posting study information on Facebook groups related to SA and trauma. Candidates received additional information over the phone. Ultimately, 21 SPs were interviewed. However, two interviews were omitted: one due to the participant’s distress and the other because the case did not meet our criteria for SA. The TPs were recruited using a similar method of posting in Facebook groups about SA therapy and therapists. Ten participants were recruited this way, and five of them referred us to eight colleagues through the snowball technique (Leighton et al., 2021). In addition, we invited two well-known therapists to participate, and yet another was recruited via the Ministry of Welfare. The final number of therapist participants was, therefore, 21. However, one interview was excluded as the participant, despite meeting the criteria of five years of experience and treating more than three male ASA survivors, held only an undergraduate degree in social work and was therefore not considered a clinician. In Israel, social workers with an undergraduate degree are eligible to treat survivors of SA; however, this qualification does not align with the professional standards required for inclusion. Thus, this exclusion ensured consistency and a focus on advanced clinical training. Eventually, 20 TPs were left in the final analysis.

All 39 interviews were audiotaped and transcribed with the participants’ signed or recorded informed consent. To ensure their anonymity, any identifying data, including names, occupations, and specific details of the assault, were either deleted from the transcripts or replaced with general information.

Semi-structured interviews were conducted with all 39 participants, each lasting from 70 to 150 min.^2^ The interviews were conversational, guided by a set of guiding questions for each group (Kallio et al., 2016). Accordingly, consistency was maintained across all participants in each group, as they were exposed to similar stimuli, ensuring uniformity across interviews for data analysis. The conversation unfolded according to the interviewees’ answers, providing an overview of the participants’ world of meaning (Kallio et al., 2016; Taylor et al., 2016). The interviews with the SPs focused on their ASA experiences, emotions, support networks, therapy, and recovery, and included several questions, among them: Can you describe your experience of seeking mental health support? What factors constrained this? What factors facilitated this? If you engaged in therapy, what factors supported or hindered your recovery? How did therapy shape your overall recovery process? What changes are needed in mental health services to better support men?

The interviews with TPs focused on their qualifications and experiences working with male ASA survivors, including their unique needs, interventions, and recovery processes, and included questions like: What symptoms have you observed? What issues are addressed in therapy with male ASA survivors? How does working with male ASA survivors differ from female ASA survivors? What interventions have you found most and least effective, and what barriers and facilitators have you encountered in therapy?

Participants could choose between face-to-face and Zoom interviews, as studies have shown that online interviews maintain rapport (Archibald et al., 2019), provide data of comparable quality to in-person interviews (Deakin & Wakefield, 2014; Mabragaña et al., 2013) and offer greater anonymity (Gray et al., 2020). A total of 12 SPs and 16 TPs chose to be interviewed via Zoom.

### Data Analysis

We used inductive experiential thematic analysis from a critical-realist perspective (Braun & Clarke, 2022; Sniewski & Farvid, 2020; Wiltshire & Ronkainen, 2021). This perspective assumes an attainable reality, while recognizing that its representations are shaped by the participants’ culture and language, among other factors (Wiltshire & Ronkainen, 2021). In addition, we applied a data-driven approach to ensure that identified themes were closely tied to the interviewees’ descriptions and that no preexisting categories were imposed by the researchers (Johnston et al., 2021; Sniewski & Farvid, 2020). Suitable for exploratory studies, this approach enables in-depth analysis, especially when existing knowledge is scarce (Sniewski & Farvid, 2020). It allowed us to interpret patterns of meaning across the data, offering insight into the participants’ lived experiences (Braun & Clarke, 2022).

The analysis followed the reflexive thematic analysis approach, emphasizing flexibility and deep engagement with the data across six stages (Byrne, 2022). First, transcripts were read and reread to immerse the researcher in the data, fostering reflective engagement and thorough understanding. Second, initial coding was conducted, with codes evolving as patterns and insights emerged. Third, the coded data were reviewed and organized into initial themes and subthemes by examining relationships among codes. Candidate themes were mapped to ensure coherence and distinctiveness, capturing the dataset’s depth and diversity. Fourth, themes were further reviewed for relevance and coherence within and across the dataset, with refinement or removal as needed to address the research questions. Fifth, themes were defined and named, with their scope and contribution to the narrative analyzed. Finally, themes were presented in a logical order, linking findings to the research questions and literature (Braun & Clarke, 2022; Byrne, 2022; Clarke & Braun, 2017).

To reflect the distinct roles and perspectives of participants, the analysis treated SPs’ first-person accounts of lived experience separately from TPs’ clinical insights. The six analytical stages were conducted independently for each group to preserve their unique viewpoints. Themes were identified within each set of interviews, and only in the final stage were they compared and integrated to create a comprehensive report. While some evaluative and reflective quotes are included, particularly from TPs, these are not treated as equivalent to experiential data. Rather, they are understood as grounded in sustained therapeutic work and used to illuminate professional meaning-making and clinical reasoning. Points of convergence between SPs’ and TPs’ perspectives are highlighted where relevant, but analytical attention is also paid to areas where experience and professional opinion diverge.

#### Trustworthiness

Our research team consisted of one doctoral student and two criminology professors, including a clinical criminologist with extensive experience in treating crime survivors. Acknowledging the gap in therapeutic focus on male ASA survivors, we aimed to explore their recovery needs and therapy dynamics, identifying facilitators and barriers. The first author, a 27-year-old female doctoral student at the time of the study, conducted and coded the interviews after undergoing specific training for interviewing SA survivors. She acknowledged potential gender and age dynamics, noting that some participants might share vulnerabilities more easily, while others could hold back due to concerns about masculinity or her young age. To establish credibility, she created a welcoming environment, allowing participants to choose locations and times for interviews. Her research background in masculinity and victimization research could have led to a focus on stigma and barriers over positive therapy experiences. To balance this, reflexive journaling and team discussions were used to interpret the data fairly.

We employed several additional strategies to increase the validity of our analysis. First, we ensured dependability (i.e., consistency of findings) by maintaining an audit trail of analytic decisions and a research journal to document coding processes, track emerging themes, and reflect on our assumptions throughout the study (Gueta & Shlichove, 2022; Stenfors et al., 2020). Second, to ensure confirmability (i.e., neutrality and minimization of researcher bias), we held regular team meetings to discuss and refine interpretations and reflect on our theoretical positions (Stenfors et al., 2020). Additionally, researcher triangulation was used to reach consensus on coding through collaborative discussions (Taylor et al., 2016). Finally, the findings were presented through rich, detailed descriptions, offering detailed contextual information supported by quotes and open conceptual analysis (Taylor et al., 2016). These steps support the transferability of the findings to other relevant therapeutic contexts (Stenfors et al., 2020).

#### Ethical Considerations

The study was approved by our university’s ethics committee, and participation was voluntary, with no compensation or incentives offered to either group. Prospective participants were informed about the study during phone calls, assured confidentiality, and signed informed consent forms acknowledging their voluntary participation and right to withdraw at any time. During interviews, they received hotline numbers and contact information for a team member with clinical expertise in victimology. The first author conducted the interviews after thorough preparation: reviewing the literature on male sexual victimization, attending training on male trauma and safe practices led by a professor, and completing additional training on masculinity and nonverbal communication from the Association of Rape Crisis Centers.

Lastly, Grammarly software was used by the authors to support grammatical editing of this manuscript during the writing process (Raheem et al., 2023).^3^

## Results

The analysis of the interviews revealed three themes: (1) facilitators of the therapeutic alliance with male ASA survivors, (2) overemphasis on trauma as a barrier to the therapeutic alliance, and (3) the in-session therapy needs of male ASA survivors. Across both groups, there was substantial agreement regarding therapeutic challenges and in-session therapy needs. At the same time, important differences in perspective also emerged. Given the distinct roles and forms of knowledge each group brings, we approached the analysis with attention to both SPs’ lived experiences and TPs’ clinical insights. While these perspectives differ epistemologically, we treated them as complementary, bringing them into dialogue to enrich our understanding of male ASA survivors’ therapeutic experiences. This integrative approach allowed us to identify areas of both convergence and divergence across the data.

### Facilitators of Therapeutic Alliance with Male Adult Sexual Assault Survivors

The first theme relates to the facilitators of building and maintaining a therapeutic alliance with male ASA survivors. This theme includes three subthemes: (a) understanding the male ASA experience—developing awareness of the distinctive nature of experiencing SA as adult men; (b) providing psychoeducation to male ASA survivors—offering information that normalizes their experiences and fosters a stronger therapeutic bond; and (c) empathetic and attentive listening—offering a non-judgmental space for them to share their experiences. The underlying focus of these three facilitators is creating and building trust with male ASA survivors.

### Understanding Male Adult Sexual Assault Experience

Nearly all the SPs described how their experience of ASA had shaped their needs and expectations from the therapy and therapists. They stated that being sexually assaulted in adulthood created unique challenges and specific needs and emotions that the therapist had to understand and address in order to facilitate a therapeutic alliance.

I feel like my emotions were stripped away. My therapist helps me understand myself... Sharing with someone who truly gets it is a huge help. I let it out, he understands the feelings and we work on it together. (Omer, SP)

I doubted so long if the sexual assault really happened. Having someone who’s willing to listen, asks what I need, and is flexible is crucial…. Being trauma-informed with male adult sexual assault survivors is key because it’s easy to look for a therapist to tell you what to do, but I want to figure it out myself. (Itamar, SP)

Similarly, the TPs emphasized the importance of understanding the survivors’ experience, with a special focus on gender roles and their intersection with adulthood and adjusting the therapy to best meet their needs. As mentioned, social expectations of masculinity and adulthood emphasize emotional stoicism, self-reliance, and strength (Levant et al., 2013; Reustle, 2021; Wood et al., 2017) and thus create a fundamental conflict between the characteristics of ASA and the therapeutic process, which involves seeking help from others. Accordingly, the TPs stressed the importance of recognizing the nuances of adult male victimization and respecting the masculine roles to which men adhere.

Sometimes “sexual assault” feels too distant to them [male ASA survivors]. We need to proceed slowly because labeling it too soon might lose them. Many resist the “victim” role, taking responsibility, saying, “I sensed my drink was spiked and could have left.” I respect their stance while affirming no one should do that to them. (Rotem, TP)

The perception is that adults don’t need help, and therapy is seen as men talking about feelings–something men aren’t supposed to do…. When men seek therapy, they need respect for the way they’ve experienced the world, the self-construct they’ve developed, their attachment patterns, and their coping mechanisms. The most basic thing I can offer them is respect–for who they are and how they’ve learned to survive. (Oron, TP)

In recognition of the need to understand the nuances of male ASA experience, many TPs have also addressed the challenges of working with male ASA survivors, particularly in relation to masculinity and age. They have discussed the intense emotional responses, especially the deep rage men often experience after ASA (Widanaralalage et al., 2023). This rage presents a significant challenge in therapy. Dorin and Gonen (TPs) relate to this issue, respectively:

Compared to women, I see rage more often among men. Rage, anger, and aggressiveness after the realization it was sexual assault.

I had patients whose aggression and anger made me afraid to sit in the room. It was unpleasant, with them often leaving the room by slamming the door and banging the wall on their way out.

In line with the importance both SPs and TPs placed on understanding the ASA, some of the SPs noted that many therapists lacked an understanding of the conflict between the masculine gender identity and SA trauma. This conflict could trigger feelings of inadequacy and weakness among survivors, which needed to be addressed:

I wanted my therapist to understand gender and political power dynamics in sexual trauma, not just how to treat trauma. Awareness of the social forces at play is crucial because admitting “I have been sexually assaulted” can be perceived as a sign of weakness and is not socially accepted. (Shoam, SP)

I felt that being sexually assaulted harmed my masculinity, and she [the therapist] couldn’t be sensitive or understanding enough… She interpreted it more from her perspective than mine. I didn’t feel compassion from her…. It seemed like my experience as an adult sexual assault victim threatened her… I did some drugs at the time, and she hyper-focused on that, not realizing it was an expression of my distress. (Eviatar, SP)

Eviatar’s excerpt indicates that occasionally, the full extent of male ASA survivors’ distress may go unrecognized by the therapist, due to differences in the ways adult men cope with the distress of SA compared to children. Men who have been sexually assaulted in adulthood would often not be seen as “ideal” victims, since being men, they are perceived as capable of defending themselves (Einat & Guter, 2024), and being adults, they are expected to avoid or handle the situation (Turanovic & Biro, 2023; Wood et al., 2017). Such perceptions and expectations, let alone the use of drugs among male ASA survivors as a coping mechanism, further distance the latter from the status of the “ideal victim,” and, thus, may reduce therapists’ empathy toward them (Einat & Guter, 2024).

### Provide Psychoeducation to Male Adult Sexual Assault Survivors

An important therapeutic aspect addressed by most SPs was learning about SA and their specific situation as male ASA survivors. According to the SPs, such learning helped them feel less isolated and gave them something to turn to. This can be described as psychoeducation, which refers to an evidence-based approach recommended for SA survivors that offers a framework for understanding experiences and adopting adaptive coping strategies (O’Doherty et al., 2023). For example, Doron (SP) stressed the importance of raising awareness for recovery: “Supporting recovery means raising awareness–letting people know this exists, that they are not alone, and that it can be treated.” Similarly, Saar (SP) shared how learning helped him process his experience: “I read a book about SA and trauma and visited the Rape Crisis Centers website, which has a page for male victims. Reading their explanations felt like someone out there understands you, and that not everyone is indifferent.”

In some cases, the SPs mentioned that their therapists played a crucial role in their acknowledgment of the SA. Therapists provided general explanations about SA, reassured them it wasn’t their fault, identified the experience as SA or rape, and emphasized that other men have faced similar situations. Roi (SP) shared how his therapist explained that SA could happen to anyone, challenging his assumptions and helping him see it was not a personal flaw.

What helped me most in therapy was the therapist telling me it can happen to anyone. It’s crucial to show other victims that it has nothing to do with personality and that it’s not their fault. It’s something that happened to them, beyond their control.

Likewise, Moshe (SP), who learned from his therapist that the legal definition of his experience was rape, asserted that this information, as well as the therapist’s guidance, helped him make better progress compared to simply discussing his experiences with the therapist.

My therapist taught me that what I went through was legally called “rape.” … She also educated me about sexual assaults. Sometimes, I’d ask her, “Please, talk more, teach me more–I build myself up better from what you say than when I speak.” I would often replay her words in my head over and over. She helped me understand so much. (Mosha, SP)

In line with the SPs’ descriptions, the TPs highlighted psychoeducation as a crucial initial stage of therapy with male ASA survivors. Learning about the ASA helps survivors normalize their experiences and empowers them. The TPs further emphasized that although they used these techniques with female ASA survivors, they employ them more often with male ASA survivors, mainly because male sexual victimization is not discussed enough. For example, Sandra (TP) shared: “I educate my patients on statistics, global trends, and the silencing of sexual assaults. This normalizing method is something I emphasize twice as much with men as with women.” Similarly, Eli (TP) shared: “The first technique I use with men is psychoeducation, normalizing their feelings … and providing references on trauma and its effects to show it’s not their fault. We also discuss the social context, including gender and sexuality.”

Sandra and Eli’s (TPs) explanations highlight that psychoeducation is particularly essential when working with male ASA survivors. Given that masculinity is fundamentally constructed in opposition to sexual victimization and that the status of adulthood further reinforces these traditional gender expectations (Levant et al., 2013; Pettersson & Carlsson, 2016; Wood et al., 2017), educating survivors regarding their sexual experiences becomes essential. Given that societal constructs, such as masculinity, are more deeply ingrained in adults (Pettersson & Carlsson, 2016; Wood et al., 2017), male ASA survivors may need further guidance to acknowledge their experiences.

### Empathetic and Attentive Listening

The SPs emphasized that one of their strongest needs in therapy was to have a therapist who genuinely listened to them. Although being heard is crucial in any therapy, for male ASA survivors, who are often overlooked due to societal expectations of masculinity and adulthood, this appears to be vital. For example, Dorel (SP) refers to the importance of being listened to: “Therapists need to first and foremost, listen and be empathetic. To be there, to listen.” Maor (SP) referred to his need to feel valued, which was fulfilled by being heard: “I think it’s crucial not to alter one’s personality, as that had already been taken by the perpetrator. Believe in the person in front of you and make them feel valued for a moment.”

Rammi (SP) similarly conveyed his need to be heard: “The therapist needs to be a vessel to hold all the hurt and listen to the pain. Working with male adult sexual assault survivors requires a bigger vessel.” His words reflect not only a desire for empathy but also a deeper hope, and perhaps fear, that the therapist can emotionally contain the intensity of his pain without being overwhelmed. This state of affairs aligns with Bion’s concept of containment (Bion, 1962), in which the therapist functions as a “container” for the client’s raw, unprocessed emotional experiences (Bion, 1962; Stubley, 2025). Through an attentive and reflective emotional presence, the therapist helps the client’s overwhelming feelings take shape and become more understandable and manageable (Stubley, 2025). For male ASA survivors, whose emotional expression often clashes with internalized ideals of masculinity and adulthood, the fear that their pain might be “too much” for another to hold adds an additional layer of emotional risk to the therapeutic encounter.

Similarly, the TPs related to empathetic listening as “therapeutic alliance” noting that the latter was often the main and sometimes the only technique necessary for working effectively with male ASA survivors in a therapeutic environment. The focus on creating a safe space for male ASA survivors to share their experiences was seen as essential for their recovery.

I don’t believe in techniques; I believe in therapeutic alliance. While I’m certified in CBT and SE [somatic experience], I don’t use them. I focus on creating a safe, trusting space…. My goal is to be with him, to make room for what he has to say and to show that I don’t judge him. I believe in empathy. (Adva, TP)

Who am I? What am I? I have theories and studies to offer, but my patient has the experience and self-knowledge, which is just as important, if not more. We will work together at his pace. The most important thing for me is to approach this with humility. What is my role? I’m here to be there for you. (Sandra, TP)

### Overemphasis on the Trauma Details as a Barrier to Therapeutic Alliance with Male Adult Sexual Assault Survivors

The second theme relates to therapists’ overemphasis on the trauma as a barrier to building and maintaining a therapeutic alliance. Some of the SPs shared that they often felt pressured by their therapists to provide more details about the ASA. This pressure was applied harshly with little regard for their immediate needs, which diminished the value of therapy. It served as a barrier to forming a therapeutic alliance with male ASA survivors, as it overlooked how ASA intersected with, challenged, and undermined their masculinity and adulthood identities. It left them feeling powerless, as the core traits they were expected to embody as men—self-reliance and strength—were stripped away (Connell & Messerschmidt, 2005; Langdridge et al., 2023). This often left them feeling unheard, misunderstood, and as if the therapist’s empathy was not genuine.

I stopped seeing one therapist after a few sessions because she didn’t truly listen when I told her what happened there [the SA]. She fixated on specific words and definitions when, in reality, I told her a story. She kept asking repetitive questions about some words I used. Overall, she wasn’t empathetic enough. (Rammi, SP)

Sometimes it felt like they [therapists] were more interested in what I was saying for their notes rather than caring about me. One of the biggest mistakes therapists make is focusing too much on the content [the details of the assault] rather than on the person. When we discuss the sexual assault, I am triggered for days. I carry this therapy, so the question is: are you having a dialogue with me, or are you treating me? (Itamar, SP)

Rammi’s and Itamar’s excerpts highlight a recurring therapeutic misattunement: therapists who focus too narrowly on the details or terminology of the SA—the content—while overlooking the survivor’s narrative as a lived, human experience. This kind of misattunement reflects a form of empathic failure—a moment when the therapist fails to recognize or respond to the client’s emotional needs, leaving the survivor feeling unseen or emotionally unsafe (Kohut & Wolf, 1978; Lerner & Jimenez, 2015). Rammi described a therapist who fixated on specific words rather than hearing the emotional arc of his story, and Itamar expressed frustration with therapists who seemed more interested in the facts than in him as a person. These moments reflect a dynamic in which the trauma is treated as a clinical object, rather than approached relationally, through the survivor’s perspective and emotional needs.

The TPs’ perspective both aligns with and differs from that of the SPs’. The similarity relates to the TPs’ understanding that they need to be subtle and gentle, not too direct or forceful when asking about the ASA, giving the survivors the space to decline sharing experiences for which they are not ready. The difference relates to the TPs’ professional stance: they need to ask further questions about the ASA, as men often do not voluntarily disclose these experiences and may need some encouragement. Danny (TP) described how he casually mentions that he specializes in male sexual victimization:

With men, it’s “don’t ask, don’t tell,” meaning if you don’t ask they will not tell you voluntarily. So, in the initial stage of therapy, I put it on the table saying that my expertise is masculinity and sexual assault among men, this is a safe space, and we can discuss it.

Sandra (TP) addressed the usage of a sign designed to help men speak up:

In my clinic, there’s a big sign that reads, “This place is a safe space to talk about sexual assaults. You are not alone. I believe you.” There is something magical about that sign because I see them sometimes looking, reading it. And while it may have been another symptom that brought them to me, I ask, “Do you see yourself in this sign?” and then they either say yes or no. (Sandra, TP)

Reluctance to share the ASA may be linked to individual and societal understanding of masculinity and adulthood which emphasize strength, self-reliance, and responsibility (Hill et al., 2023; King et al., 2021; Levant et al., 2013; Turanovic & Biro, 2023). This means that men who have experienced ASA find it difficult to reveal details that place them at a disadvantage or portray them as having failed to live up to masculine and adult ideals. Hence, many of the TPs stressed the importance of providing a safe space. Underscoring this point, Libbi (TP) noted that sharing the ASA left the survivors feeling helpless.

From what I see, when you’re an adult male, you can’t be in a vulnerable state. You see them questioning: “Who am I? Can I be part of society if I’ve been sexually assaulted?” So they tell themselves, “I should just keep it quiet and be a man.” When it comes to talking about sexual assault, some never reveal it, but I suspect, they never say it, and we’re still in that process. Others share it really quickly, just after a few sessions. I think it’s because, on some level, I have become a part of their life. And once we established safety, once they sensed it was safe, it just poured out.

Gonen (TP) also noted men’s need for a safe space, by stating that as the therapist he is sometimes the only one who holds their ASA story and listens to them without judgment:

For me, it’s crucial to be present and sometimes the only witness to this secret, to echo it correctly. Sometimes, I’m the only one who knows my patients’ story, the only one who can hold it, and the only one they can talk to without feeling shame, fear, or dread.

### Male Adult Sexual Assault Survivors’ In-Session Therapy Needs

The third theme relates to the specific therapy needs of male ASA survivors. It includes two subthemes: (1) a therapeutic approach that prioritizes the here and now rather than the past; and (2) breaks outside the clinic.

#### A Therapeutic Approach that Prioritizes the Here and Now Rather than the Past

In line with the survivors’ hesitations about sharing their SA, the SPs also mentioned that, unlike therapy and therapists who often focused too heavily on the past, they preferred approaches that better addressed their current needs. According to Eviatar (SP): “Psychological therapy felt very long and often seemed to focus on the past rather than the present.” Likewise, Soham (SP) explained: “Often, it [therapy] feels like it just digs into memories and circles around a certain narrative. What I need is to feel it in my body–my body is constricted, my breath is cut off, and I freeze.” Levi (SP) reflected on how therapy should approach sensitive topics in a way that prioritizes the here and now over the details:

It’s important to slow down and not rush people. In the first sessions, there’s no real sense of safety yet–and then you expect them to share everything? That pushes them away. You need to go slow. Especially with something this sensitive, leave the details alone, they don’t matter all that much. Telling the story matters more than the story itself.

Similarly, the TPs voiced the importance of being in the moment with men and not always trying to get into deeper issues. Oron (TP), for example, emphasized the importance of attending to men’s concrete daily needs without necessarily focusing on sensitive areas.

I think many men drop out of therapy because it doesn’t suit them; we need something different. I’m still learning, but men need a practical approach, not just talking. It’s important to allow concrete discussions about current events in their lives. For example, when he talks about his job or a place he visited with his girlfriend, listen and don’t interrupt and push for deeper issues.

Hagi (TP) described how he gives patients space and follows their pace:

Sometimes they [adult men] jump to the details, and I say–wait, slow down. I want to focus on the present moment, and only then, when they’re ready, we go back. What matters is what they bring, and how they cope. I try to create a unique setting with each person, ask what they need, and let them know–I’m here, right here, right now with you.

Taken together, the perspectives of both SPs and TPs reflect a broader need among male ASA survivors for therapeutic approaches that prioritize the present moment over an in-depth exploration of past trauma. The emphasis on practicality suggests that, for some men, therapy feels safer and more effective when grounded in their current life circumstances rather than when it immediately explores emotionally intense material.

An additional aspect raised by the TPs concerned the physical context of therapy and how it may shape the therapeutic process with male ASA survivors.

#### Breaks Outside the Clinic

Many of the TPs described alternative approaches that involved taking breaks or conducting parts of the therapy outside the clinic setting. While many SPs expressed a general desire for something different due to the perceived limitations of traditional therapy formats, it was the TPs who articulated these needs through concrete, practice-based adaptations.

Some of the TPs supported such an approach, given its flexibility and informality, which helped male ASA survivors connect to the therapeutic process and build a better therapeutic alliance. They also stressed that this approach was more commonly used with men, particularly ASA survivors:

I notice that traditional post-trauma techniques aren’t very effective for men sexually victimized as adults. One helpful practice is taking breaks outside, like smoking on the balcony, going for walks, or sitting on a bench. These moments of stepping out for fresh air are relaxing. I tried relaxation exercises, but many men struggled with them. (Dorin, TP)

I had a patient with whom therapy was stagnant until we started going for walks outside. The moment we stepped out, everything changed…. Another patient couldn’t handle sitting in the therapy room, so we met in the garden, sitting on the grass and talking while playing with branches and discussing. Working in nature makes a big difference. (Reshef, TP)

However, a few TPs highlighted the importance of preserving the clinic-based setting.

I believe in keeping the setting; this is a technique I strongly believe in and I think should be kept. If the setting is the outside, it is okay too, but the setting needs to remain. Eventually, this is what keeps the therapy. (Yanir, TP)

I feel that the clinic is a very protective place and also very important. I feel that situations where you leave the clinic can be very good at times but can also be very confusing. And, certainly with victims of sexual trauma, I am very protective of the setting. (Gidi, TP)

## Discussion

The present study aimed to analyze the therapeutic alliance and in-session therapy needs of male ASA survivors, from their own and from sexual trauma therapists’ perspectives. Although previous research has stressed the importance of gender-sensitive approaches when working with male SA survivors (Elkins et al., 2017; Langdridge et al., 2023), this is, to the best of our knowledge, the first study to explore the formation of the therapeutic alliance and in-session therapy needs among male ASA survivors, while specifically considering the role of both gender and age at the time of assault in shaping these therapeutic processes. The discussion presented here synthesizes the study’s findings into broader concepts, connecting them to the cultural, gendered, and age-related dimensions that shape both the therapeutic process and the survivors’ in-session therapy needs. It is important to note, however, that these findings reflect a predominantly heterosexual understanding of masculinity and may not fully capture how queer survivors experience trauma, identity, and therapy.

Our first key finding relates to the nuances of how a therapeutic alliance is built with male ASA survivors. The study identified three key facilitators of therapeutic alliance: (1) being mindful of the differences in male ASA survivors’ experience in relation to masculinity and adulthood, (2) usage of psychoeducational bonding for normalizing male ASA experiences, and (3) present-focused listening. These elements were perceived by both the SPs and the TPs as essential in building and facilitating therapeutic alliance with male ASA survivors.

In line with previous calls to adapt therapy for men by addressing masculine gender roles (Bedi & Richards, 2011; Elkins et al., 2017; Rapsey et al., 2020), the underlying principle of the three facilitators revealed in our study is sensitivity to the unique situation experienced by men sexually assaulted in adulthood. Previous studies have found that the clash between victimhood and cultural expectations of masculinity generates profound feelings of shame, humiliation, isolation and fear that are similar between survivors of CSA and ASA (Guter et al., 2025; Langdridge et al., 2023; PettyJohn et al., 2022). However, our findings introduce an additional layer: the role of adulthood. Male ASA survivors face a dual challenge: reconciling their victimization not only with masculinity ideals but also with their adult identity, which is often associated with independence, control, and responsibility (Hill et al., 2023; Turanovic & Biro, 2023; Wood et al., 2017). This layered conflict complicates their therapeutic experience and requires acknowledgment in clinical settings. The three facilitators identified in this study reflect the need to address this complexity. When therapists demonstrate awareness of these intersecting identities, offer relevant psychoeducation, and show a genuine willingness to listen, they foster trust. Given that trust is a fundamental component of the therapeutic alliance (Ardito & Rabellino, 2011; Howard et al., 2022), it serves as a facilitator thereof.

Moreover, based on these three facilitators, we identified two complementary forms of trust that capture how the therapeutic alliance was experienced by male ASA survivors: empathic trust and competent trust. Empathic trust refers to the trust that develops when survivors feel that the therapist attunes to the specific reality of being sexually assaulted in adulthood. It is built when therapists understand that the survivor’s feelings, struggles, and identity are shaped by this particular experience, and respond with genuine presence, validation, and attentive listening. Survivors come to trust their therapist because they truly “get” the meaning of being assaulted as an adult man and allow space for it without judgment. This form of trust was fostered through the first and third therapeutic facilitators: recognition of the unique nature of ASA and present-focused listening. *Competent trust,* in contrast, emerges when survivors gain confidence in the therapist’s professional knowledge to address the particular challenge of male ASA. This form of trust is rooted in the therapist’s capacity to explain, teach, and offer clarity about what the survivor is experiencing. Through psychoeducational bonding—the second facilitator—therapists provided survivors with language, frameworks, and validation that made their internal experiences feel understandable and manageable. Competent trust allows the survivor to feel that the therapist is not only empathetic but also equipped to provide effective, informed intervention.

The two forms of trust are especially important for male ASA survivors, as they address core emotional needs and offer validation for an experience that is often overlooked (Barnard, 2018). This is particularly vital given that SA recovery is shaped by social and cultural factors, including gender and age, which influence the recognition and support survivors receive (Gueta, 2022). Our study found that both forms of trust can be disrupted when therapists do not engage in present-focused listening—the third facilitator of the therapeutic alliance. Many SPs emphasized the importance of feeling genuinely heard by their therapists. Conversely, they described how being perceived solely as SA survivors, and encountering therapists who listened without authentic engagement—such as focusing on the trauma rather than the person—undermined the fragile trust between them. This dynamic created a significant barrier to building a strong therapeutic alliance. Therefore, we believe that in treating male ASA survivors, therapists must engage in dialogue that respects emotional boundaries, recognizes the person beyond the trauma, and prioritizes the survivor’s well-being over clinical detachment.

In the Israeli context, while Israel shares many similarities with other Western countries (Lowenstein-Barkai, 2021) its cultural values surrounding masculinity are uniquely shaped by mandatory military service and the ethos of the fighter and warrior (Levy & Sasson-Levy, 2008). These ideals emphasize strength, resilience, and invulnerability (Gilbar et al., 2019), potentially intensifying the internal conflicts experienced by male ASA survivors. Additionally, adulthood in Israel is closely tied to fulfilling military and familial roles (Gueta & Shlichove, 2022; Levy & Sasson-Levy, 2008), which can compound feelings of shame or failure for survivors who perceive their trauma as a barrier to meeting these expectations. By fostering both empathic and competent trust, therapists can help male ASA survivors navigate these complex dynamics. For example, empathic trust allows therapists to recognize and address how the cultural ideals of strength and responsibility intersect with survivors’ emotional struggles, while competent trust reassures survivors of the therapist’s ability to guide them through their recovery. These forms of trust not only provide validation but also create a culturally sensitive framework that supports survivors in reconciling their personal experiences with societal expectations.

Our second key finding concerns three in-session therapy needs of men who have been sexually victimized as adults. These needs, hitherto not fully addressed by current therapeutic approaches, are (1) psychoeducation, (2) reducing focus on past experiences and emphasizing the present, and (3) leaving the therapy clinic for short breaks. With regard to psychoeducation, it emerged in our study both as a facilitator of the therapeutic alliance and a critical in-session therapy need for male ASA survivors. The SPs emphasized the importance of having a therapist who understands their situation as adult men and knows how to approach it and educate them. Correspondingly, the TPs highlighted the significance of psychoeducation in therapy for male ASA survivors, indicating that it might be used more frequently with male survivors than with female survivors. Psychoeducation is recognized as an evidence-based approach (O’Doherty et al., 2023), and is also recommended for use with men in therapy overall (Beel et al., 2019; Mahalik et al., 2012).

At the same time, it is important to acknowledge that this approach may not align with all therapeutic orientations. In more experiential or insight-driven approaches (e.g., psychodynamic, psychoanalytic, or emotion-focused therapies) directive or information-based interventions like psychoeducation may be seen as potentially intrusive or misaligned with the therapeutic process (Busch & Auchincloss, 2018; Gokarakonda & Jensen, 2022; Marren et al., 2022). Despite this potential tension, the current findings underscore psychoeducation’s particular value for male ASA survivors. It served not only as a tool for conveying information but also as a means of recognizing, validating, and normalizing their experiences. In this context, psychoeducation appears to help bridge the gap created by masculine and adult norms that make it difficult for men to articulate or even acknowledge their victimization (Barnard, 2018; Hill et al., 2023; Reed et al., 2020). Thus, our findings suggest that psychoeducation, when used flexibly and with sensitivity, can play a unique role in supporting emotional expression and connection in therapy with male ASA survivors.

With regard to reducing the focus on past experiences and emphasizing the present, SPs shared how therapy often pulled them back into the past when trying to move forward and expressed their desire for a more present-focused approach. In a similar vein, the TPs claimed that addressing daily issues of male ASA survivors is extremely important, at least as working through trauma, since it indicates that someone is genuinely listening to their everyday experiences and provides them with concrete solutions. Highlighting tangible matters while laying less emphasis on emotions may help male ASA survivors, since they often find the sharing of emotions challenging (Hogan, 2022; Seidler et al., 2018). Moreover, a present-focused approach that emphasizes current strengths and practical solutions and aligns with socially reinforced masculine gender roles that emphasize action and problem-solving (Levant et al., 2013; Mahalik et al., 2012), may help male ASA survivors alleviate feelings of losing control and power. A present-focused approach aligns with gender-sensitive therapy, which in this context calls for attention to both masculine gender norms and the expectations associated with adulthood, which may shape how male survivors engage in the therapeutic process (Bedi & Richards, 2011; Elkins et al., 2017; Rapsey et al., 2020).

Finally, the TPs were ambivalent about the idea of leaving the setting for short breaks. Whereas some argued that male ASA survivors have a genuine need to take occasional breaks, others argued that remaining in one therapeutic setting was significant for their therapy. Male SA survivors often find it challenging to discuss their feelings or engage fully in therapy (Bedi & Richards, 2011; Hogan, 2022; Seidler et al., 2018), particularly when therapy requires a sustained focus on emotional and traumatic experiences—areas that may conflict with internalized masculine norms. Given that the survivor participants in our study emphasized difficulty speaking about trauma and a preference for focusing on present-day experiences, it is reasonable to assume that continuous trauma-focused dialogue within a fixed setting may intensify discomfort. For some, the pressure to speak about the trauma in a confined space may lead to emotional overload and heightened vulnerability while trying to appear composed. Additionally, therapy demands prolonged emotional attention, which can lead to cognitive and emotional fatigue (Matise & Price-Howard, 2020). In this context, transitioning to an outdoor or alternative space may offer restorative benefits. As noted in the literature on nature-based interventions, environments that gently capture attention can help reduce mental fatigue and support emotional regulation (Matise & Price-Howard, 2020). In this way, our findings align with previous research suggesting that male SA survivors may benefit from greater flexibility in therapeutic structure and setting (Bedi & Richards, 2011; Gamache et al., 2025; Moor et al., 2022; Rapsey et al., 2020).

While breaks from the therapy room may offer male ASA survivors a less emotionally intense and more approachable therapeutic experience, they also raise concerns about reinforcing avoidance behaviors. Elder et al. (2017) highlight how masculine norms often lead men to adopt coping strategies that appear action-oriented but serve to avoid emotional processing. Similarly, Neilson et al. (2020) found that traditional masculinity ideologies, particularly those emphasizing emotional control and stoicism, can hinder trauma treatment by encouraging avoidance of vulnerability and emotional disclosure.

Following this line of reasoning, maintaining a consistent therapeutic setting may benefit male ASA survivors, mainly since it provides safety and privacy. Previous research has portrayed the therapy room as a healing space that defines therapist-client roles, offers safety and privacy, and plays a key role in building the therapeutic alliance (Sinclair, 2021). For male ASA survivors, this may be especially important considering that they are seen as individuals who have failed to realize both hegemonic masculinity (Einat & Guter, 2024; Vandello et al., 2008) and adulthood ideals (Hill et al., 2023; Wood et al., 2017). This juxtaposition could intensify their trauma which further deepens their need for a safe and consistent environment.

To conclude, this study identifies key factors in building a therapeutic alliance with male ASA survivors and their specific in-session therapy needs. It emphasizes how masculinity and adult age shape the therapeutic process and suggests three key facilitators: recognizing their unique experiences related to gender and age, using psychoeducational bonding to normalize their experiences, and practicing present-focused listening. Additionally, the study highlights key strategies for therapy with male ASA survivors, including psychoeducation, prioritizing the present moment, and considering incorporating breaks outside of the clinical setting while prioritizing the well-being of the patients and ensuring anonymity.

### Limitations and Future Research Directions

Despite its contributions, this study suffers from several limitations. First, the sample lacked sexual-orientation diversity, as nearly all SPs identified as heterosexual. This limits generalizability, as therapeutic needs may differ across sexual orientations (Ralston, 2020; Wingender & Olesen, 2024). Future research should address this gap to inform more inclusive therapeutic practices. Second, eight of the 19 SPs had experienced both CSA and ASA, which may have shaped their alliance formation and in-session therapy needs. Future research should explore the experiences of ASA survivors without a history of CSA. Third, the study includes indirect information provided by TPs, as their accounts reflect interpretations of their clients’ experiences (Widanaralalage et al., 2023). While these professional perspectives should be interpreted with caution, this limitation is partly addressed by combining the viewpoints of both survivors and therapists. This dual-source approach introduces its own complexity, as each group offers a different type of data. However, the study did not treat these perspectives as equivalent but as complementary in order to mitigate this issue. In addition, although the TPs had diverse backgrounds and therapeutic approaches, the analysis did not account for differences in therapeutic orientation, in line with the study’s aims. Future research should explore how therapeutic orientation shapes therapists’ perceptions of the alliance and in-session needs of male ASA survivors.

Additionally, the recruitment process primarily relied on Facebook groups, which introduced potential self-selection bias. To address this, we posted in 67 groups of different sizes to maximize diversity. Future studies should explore broader recruitment strategies, such as partnerships with clinics and organizations, to ensure a more representative sample. Furthermore, as therapy breaks were a divisive topic among TPs, further research is needed to examine their specific effect on male ASA survivors. This could clarify whether and how such breaks address survivors’ unique therapeutic needs. Lastly, our study did not differentiate between types of assault (e.g., being penetrated vs. FTP). These dynamics may carry distinct implications given masculinity norms that associate activity with strength and passivity with weakness (Einat & Guter, 2024; Weare, 2018). Future research could explore how such nuances shape therapeutic dynamics.

### Clinical Implications

Based on the present findings, we recommend that therapists be mindful of how gender and age shape the therapeutic experience of male ASA survivors by adopting a more present-focused approach. This includes beginning sessions by exploring the client’s current life (e.g., work, relationships, or daily challenges) rather than immediately focusing on traumatic events. Therapists can invite clients to reflect on recent emotional experiences, challenges in their daily functioning, or shifts in mood and behavior, creating space for therapeutic engagement that feels manageable, present-focused, and less emotionally overwhelming. In addition, given the importance of psychoeducation highlighted in this study, we suggest introducing it early in the therapeutic process to help reduce shame and normalize the client’s responses. This may involve addressing common myths about male sexual victimization, offering relevant statistics, or discussing the societal pressures that contribute to silence and stigma. These messages can be delivered through open conversation or supportive materials, depending on the client’s preferences and readiness. This approach may ease the emotional intensity many men feel in therapy by offering structure, validation, and flexibility.

Finally, we encourage clinicians to consider incorporating short, intentional breaks—whether by stepping outside briefly or having some sessions outside—as a means of supporting emotional regulation. These breaks should be negotiated collaboratively and implemented with careful attention to the client’s comfort, needs, and confidentiality. We recognize that therapy is not universally applicable, and individual needs often outweigh gender norms. However, we believe that following these recommendations will enhance the therapeutic relationship and address male ASA survivor in-session therapy needs, ultimately leading to more effective therapy for them.

## Footnotes

1 Out of the 16 participants who engaged in therapy, most did so for reasons not related to the SA, which was only discussed later. It is possible that those with prior therapeutic experience expressed themselves with greater awareness or in terms shaped by clinical discourse. Nonetheless, the thematic analysis showed that similar core themes were raised across participants, regardless of whether they had attended therapy.

2 All interviews with the SPs and TPs were conducted in Hebrew and meticulously translated into English by the authors. The translation process was primarily carried out by one author, with oversight and review from the other two authors to ensure accuracy of meaning and context. All three authors are fluent in both Hebrew and English, which facilitated precise translation and contextual fidelity. The translations were thoroughly proofread to enhance clarity and maintain alignment with the original data.

3 Grammarly was used to maintain grammatical accuracy without generating any content. Previous studies have shown it as an effective tool that improves clarity and grammatical accuracy when used alongside careful judgment (Raheem et al., 2023).
